# Psychotherapy for Ketamine’s Enhanced Durability in Chronic Neuropathic Pain: Protocol for a Pilot Randomized Controlled Trial

**DOI:** 10.2196/54406

**Published:** 2024-04-17

**Authors:** Akash Goel, Bhavya Kapoor, Hillary Chan, Karim Ladha, Joel Katz, Hance Clarke, Janneth Pazmino-Canizares, Zaaria Thomas, Kaylyssa Philip, Gabriella Mattina, Paul Ritvo

**Affiliations:** 1 Department of Anesthesia and Pain Medicine St Michael’s Hospital Toronto, ON Canada; 2 Department of Anesthesiology and Pain Medicine University of Toronto Toronto, ON Canada; 3 Department of Medicine Royal College of Surgeons in Ireland Dublin Ireland; 4 Temerty Faculty of Medicine University of Toronto Toronto, ON Canada; 5 Institute of Health Policy, Management and Evaluation University of Toronto Toronto, ON Canada; 6 Department of Psychology York University Toronto, ON Canada; 7 Department of Anesthesia and Pain Management Toronto General Hospital Toronto, ON Canada; 8 Centre for the Study of Pain University of Toronto Toronto, ON Canada; 9 School of Kinesiology and Health Sciences York University Toronto, ON Canada

**Keywords:** 3-arm parallel group, cognitive behavior therapy, ketamine hydrochloride, pain intensity, pain interference, psychotherapy, randomized controlled trial

## Abstract

**Background:**

Chronic pain affects approximately 8 million Canadians (~20%), impacting their physical and mental health while burdening the health care system with costs of upwards of US $60 billion a year. Indeed, patients are often trialed on numerous medications over several years without reductions to their symptoms. Therefore, there is an urgent need to identify new therapies for chronic pain to improve patients’ quality of life, increase the availability of treatment options, and reduce the burden on the health care system.

**Objective:**

The primary objective of this study is to examine the feasibility of a parallel 3-arm pilot randomized controlled trial whereby patients are randomized to either intravenous ketamine alone, cognitive behavioral therapy (CBT) and mindfulness meditation (MM) training (CBT/MM), or the combination of intravenous ketamine and CBT/MM. The secondary outcome is to assess the durability and efficacy of combination intravenous ketamine and CBT/MM for treatment of chronic pain as compared to CBT/MM or intravenous ketamine alone (assessed at week 20 of the study).

**Methods:**

This is a single-center, 16-week, 3-arm pilot study that will take place at the Chronic Pain Clinic at St. Michael’s Hospital, Toronto, Ontario, which receives 1000 referrals per year. Patients will be enrolled in the study for a total of 20 weeks. Participants who are allocated CBT/MM therapy will receive remote weekly psychotherapy from week 1 to week 16, inclusive of health coaching administered through the NexJ Health Inc (NexJ Health) platform. Patients who are allocated ketamine-infusion therapy will receive monthly ketamine infusion treatments on weeks 2, 7, and 12. Patients who are allocated ketamine+CBT/MM will receive weekly psychotherapy from weeks 1 to 16, inclusive, as well as ketamine infusion treatments on weeks 2, 7, and 12. We will be assessing recruitment rates, consent rates, withdrawal rates, adherence, missing data, and adverse events as pilot outcome measures. Secondary clinical outcomes include changes relative to baseline in pain intensity and pain interference.

**Results:**

As of November 1, 2023, the recruitment process has not been initiated. Given the recruitment, consent, and intervention target of 30 participants for this feasibility study, with each patient undergoing monitoring and treatments for a course of 20 weeks, we expect to complete the study by December 2025.

**Conclusions:**

This study assesses the feasibility of conducting a 3-arm randomized controlled trial to examine the effects of ketamine administration with the concurrent use of CBT/MM in a population with chronic neuropathic pain. The results of this pilot randomized controlled trial will inform the development of a larger-scale randomized controlled trial. Future studies will be aimed at including a sufficiently powered sample that will inform decisions about optimal treatment calibration and treatment effect duration.

**Trial Registration:**

ClinicalTrials.gov NCT05639322; https://classic.clinicaltrials.gov/ct2/show/NCT05639322

**International Registered Report Identifier (IRRID):**

PRR1-10.2196/54406

## Introduction

Chronic pain affects approximately 8 million Canadians (~20%) and profoundly impacts their physical and mental health. The broad ranging consequences of this disease are evidenced by estimated health care system costs upwards of US $60 billion a year [1]. In the United States, chronic pain also affects 1 in 5 people, and the annual cost is estimated as greater than US $0.5 trillion [2]. Indeed, chronic pain is associated with poor quality of life, psychiatric symptoms, and mobility restrictions. It has significant physical, functional, social, and economic impacts at the individual and system levels. Current pain therapies aim to decrease pain to manageable levels. In most cases however, chronic pain cannot be completely eliminated. Patients are often trialed on numerous medications without adequate relief, and therapies are often limited by their lack of durability and adverse effects. There is an urgent need to identify new therapies for chronic pain to improve patients’ quality of life and the availability of treatment options, while reducing health care system burdens.

Ketamine is an N-methyl-D-aspartate receptor antagonist which results in pain reduction. It is also associated with reductions in opioid-induced hyperalgesia and opioid consumption [3]. At high doses, it is an agonist at the mu receptor, the D2-R, and L-Type voltage-gated calcium channel. Current evidence supports intermittent ketamine therapy for neuropathic pain. Double-blind randomized controlled trials (RCTs) investigating the use of ketamine for neuropathic pain have demonstrated clinically significant reductions (25% to 43%) in chronic pain versus control conditions [3]. However, ketamine appears to have limited durability as studies have shown short term reductions in neuropathic pain secondary to spinal cord injury and fibromyalgia but no long-term benefits [4,5]. Although timelines and follow-ups vary significantly, the lack of durability is consistent across all ketamine infusion regimens studied [6]. Indeed, a systematic review by Cohen et al [3] indicated a lack of evidence for durable benefits, affirming the need for adjunctive therapies that improve durability and improve outcomes.

Internet-delivered cognitive behavioral therapy (CBT) for chronic pain, depression, and anxiety is associated with significant reductions in pain disability, symptoms of depression, and pain catastrophizing at 1 year follow-up [7]. Nonetheless, an integrative review [8] concluded CBT research must further address longer-term efficacy (>12 months). This same review highlighted the significant variations in the duration and total dose hours of CBT interventions and delivery methods (remote, group, or individual). Despite variations, existing studies and emergent guidelines support the synergistic effects of pharmacological therapies strategically combined with CBT-based strategies [7].

The primary objective of this trial is to study the effects of concurrent ketamine and CBT for chronic neuropathic pain relief. Biological and neurocognitive factors suggest that concurrent pharmacotherapy and psychotherapy can result in more durable and effective analgesic responses than either intervention alone. Ketamine administration induces physiological changes during and after delivery that result in powerful experiential responses with important psychological and emotional impacts [9]. The physiological changes associated with ketamine therapy are likely to influence important habitual thinking and behavior patterns. Studies suggest that ketamine alters functional brain connectivity, even to the point of disrupting spatiotemporal patterns of neural activity and increasing functional communications between brain regions typically isolated [9].

Despite the biologic plausibility of ketamine-assisted psychotherapy being an effective cotreatment for chronic neuropathic pain, there is a lack of rigorous evidence supporting its use. Our group conducted a systematic review to examine existing evidence on concurrent ketamine dosing and psychotherapy [10]. This knowledge synthesis showed substantial methodologic variations across 17 studies. The most common indications studied included posttraumatic stress disorder (6 studies), major depressive disorder (6 studies), substance use disorder (6 studies), and obsessive compulsive disorder (2 studies). Only 2 publications (a case report and a case series) examined continuous multiday ketamine infusions with concurrent psychotherapy in individuals with chronic pain. Ocker et al [11] described a 5-day continuous ketamine infusion regimen combined with CBT to successfully taper opioids in a patient with complex regional pain syndrome who was taking 330 mg of daily morphine equivalents. They attribute the sustained results to an observed synergism between ketamine and the outpatient CBT protocol. Keizer et al [12] described a case series of 11 patients who received ketamine-enhanced psychotherapy for comorbid neuropathic pain and posttraumatic stress disorder. A 96-hour ketamine infusion was delivered with daily bedside psychotherapy. Although it resulted in a moderate but clinically meaningful reduction in pain symptoms, the brief follow up of 5 days precluded in-depth examination of the potential synergism.

This systematic review also revealed no evidence that a single protocol or therapy strategy was decidedly more beneficial than any other. To date, no RCT has combined ketamine and psychotherapeutic intervention for patients with chronic pain. The results of this review highlight the poor methodological quality of existing studies and need for higher quality evidence on the potential impact of combined interventions. By means of this pilot study, our intention is to determine whether a customized CBT along with mindfulness and meditation (MM; ie, CBT/MM) psychotherapy regimen can be effectively and safely integrated with a ketamine-induced state in a chronic neuropathic pain sample.

The Psychotherapy for Ketamine’s Enhanced Durability in Chronic Neuropathic Pain study has received partial funding from the Canadian Pain Society in partnership with the Pfizer Early Career Investigator Award as well as the St. Michael’s Hospital Association Innovation Fund.

## Methods

### Aim

The primary objective of this study is to enroll 30 participants with chronic neuropathic pain and assess the feasibility of conducting a 3-arm parallel group pilot RCT. The secondary aim of the study is to examine whether the combined effects of ketamine and CBT/MM (as delivered through the NexJ Health Inc platform by trained navigator-coaches) can reduce pain intensity and pain interference by week 20 of the study (ie, limited efficacy at week 20).

### Recruitment and Randomization

Recruitment will occur at the Chronic Pain Clinic at St. Michael’s Hospital, Toronto, Ontario. The clinic receives approximately 1000 referrals per year. An estimated 3% of these referrals would be eligible for inclusion in this trial.

A staff member who does not belong to the study team will be provided with the instructions to use a remote random number generator, the “Sealed Envelope” website to generate an allocation sequence at random. The allocation list will be sent to the Research Pharmacy staff at St. Michael’s Hospital directly, without the knowledge of the study team, to maintain the blindness. After participant enrollment, the pharmacy staff will randomize the patient and inform a delegated research team member as to the allocated intervention arm. The delegated unblinded research team member will then notify the patient which arm they have been randomized to and will coordinate with the anesthesiologists and psychologists, who will be unblinded, to schedule in-person study visits on weeks 2, 7, and 12 according to the randomized treatment arm. For patients randomized to Arm 1 or 3, the Research Pharmacy staff will prepare ketamine-hydrochloride for weeks 2, 7, and 12. The outcome assessors are the only research staff to remain blinded to patient treatment (currently, we only have 1 delegated outcome assessor).

Participants will be randomized to receive either (1) ketamine-hydrochloride infusions alone, (2) CBT/MM alone, or (3) a combination ketamine-hydrochloride infusion and CBT/MM.

Participants will be eligible for this study as per the inclusion and exclusion criteria listed (Textbox 1).

**Inclusion and exclusion criteria regarding participant eligibility for PYSKED-NP.**

**Inclusion criteria**

- Aged 18 years or older.
- Diagnosis of chronic neuropathic pain as determined by a pain specialist with moderate to severe neuropathic pain as per ID pain questionnaire, with mean pain scores >3 on an 11-point (0-10) Numeric Rating Scale, in the 7 days preceding inclusion.
- For participants of childbearing potential, use of highly effective or double-barrier methods of contraception. Abstinence is acceptable if it is the preferred and usual lifestyle of the participant.
- Capacity to provide informed consent

**Exclusion criteria**

- Patients aged younger than 18 years.
- Current or lifetime history of schizophrenia, psychotic disorder, bipolar disorder, or borderline personality disorder.
- Known history of hypersensitivity or allergy to ketamine-hydrochloride.
- Current history of dissociative disorders.
- Current concomitant use of theophylline or aminophylline.
- Current elevated intracranial pressure.
- Pregnancy or ongoing breastfeeding in female participants.
- Concomitant active substance use in the 6 months preceding enrollment (amphetamines, alcohol, and ketamine).
- Contraindication to receiving ketamine-hydrochloride (eg, current or lifetime history of cerebrovascular accident, current significant hypertension [systolic blood pressure higher than 160 mm Hg in combination with or with an isolated diastolic blood pressure higher than 100 mm Hg], and current severe cardiac decompensation [eg, presence of dyspnea, peripheral edema, elevated jugular venous pressure, hepatomegaly, pulmonary rales, and pleural effusions]).

### Withdrawal Criteria

A participant will be withdrawn if the participant presents with a severe adverse event or unknown allergic reaction during the first administration of ketamine-hydrochloride or if the participant wants to withdraw from the study for any reason. The participant will receive the necessary treatment to alleviate any untoward events and will be monitored by the study physician until judged appropriate for discharge. Participants will be followed up on the following day to ascertain more details on their condition. Data from a participant who has been withdrawn will be collected up to the day following their withdrawal to ensure that the participant has no more complications. If a participant withdraws from the study at any time, the reasons for withdrawal will be collected and documented as a feasibility study outcome.

Since this is a feasibility study without a specific sample size required to analyze any outcome, participants will not be replaced or added.

### Intervention

#### Intervention Description

Participants will be randomly assigned to one of 3 arms. Arm 1: Ketamine-hydrochloride infusions alone, Arm 2: CBT in combination with MM (CBT/MM), or Arm 3: Ketamine-hydrochloride infusions in combination with CBT/MM.

Ketamine-hydrochloride will be delivered intravenously for patients in Arm 1 and Arm 3. For this study, 1 mg/kg of ketamine intravenous will be dosed by total body weight and administered over 2 hours up to a maximum dose of 100 mg as per our local institutional protocol.

#### Arm 1: Ketamine-Hydrochloride Infusion Alone

Patients in Arm 1 receiving a ketamine-hydrochloride infusion alone will receive ketamine infusions on weeks 2, 7, and 12 of the study. After the study intervention, participants will have their vital signs and adverse events monitored for one hour. The anesthesia provider will be responsible for assessing the participant before discharge. Participants will not be allowed to drive after the intervention; therefore, measures will be taken to ensure the participant has an adequate plan of transportation (ie, a companion or suitable transportation).

For safety reasons, the anesthesia provider administering the medication will not be blinded. However, the study personnel designated to collect the outcome data will be unaware of the treatment assignment. Patients will not be blinded to their therapy for the purposes of this feasibility study.

#### Arm 2: CBT/MM Alone

Participants in Arm 2 receiving CBT/MM alone will receive weekly CBT/MM treatment in collaboration with NexJ Health Inc and supplemented by the designated psychotherapist trained for and experienced in previous studies [13]. About 16 hours of CBT/MM therapy will be delivered over 16 weeks. Participants will be assigned to a 3-hour CBT/MM workshop remotely, to be administered by the delegated psychotherapist. The CBT-MM programming will combine exposure to smartphone- and computer-accessible workbooks with phone-based mental health counseling (16 hours in 16 weeks) that coordinates with ongoing software interactions (eg, secure text messaging) [14,15].

The software for delivering the CBT/MM therapy is a standalone platform, called “Connected Wellness Platform,” developed by NexJ Health Inc. Connected Wellness Platform is a customized, secure (2-level log-in with 128-bit encryption) interface accessible through both a smartphone and a computer.

#### Arm 3: Ketamine-Hydrochloride Infusion in Combination With CBT/MM

Finally, participants in Arm 3 receiving ketamine infusion+CBT/MM will receive both the standard CBT/MM and ketamine-hydrochloride infusion protocols as described above. Participants in this arm will have psychotherapy delivered concurrently with their ketamine infusions on weeks 2, 7, and 12 in person by the delegated psychotherapist. Participants in Arm 3 will also receive 16 weeks of CBT/MM remotely.

#### Hypothesis

In line with the primary aim of this study, this pilot study will prove to be feasible for a large-scale clinical trial. We expect that we will be able to successfully meet our recruitment target (30 participants) within the 1-year time frame of the study and expect this study withdrawal rate to be less than 10% (3/30 patients). Feasibility of this study will be determined by the following set variables and cutoff percentages; recruitment success with the specific aim to recruit all necessary patients within the first year of the trial period, adequate consent rate (30/100, >30% of eligible patients consent to participate in the study), intervention adherence as define by the percentage of patients who complete all medication and psychotherapy visits (27/30, >90%), percentage of patients who withdraw from the study (ie, due to side effects; 6/30, <20%), percentage of patients with incomplete pain intensity and pain interference data at 1 month, (eg, withdrawal due to side effects, loss to follow up, or missing data; 3/30, <10%), and rate of adverse outcomes. We do not foresee any issues arising with the protocol of the study or the delivery of each arm.

While not powered to examine these effects, we hypothesize that patients allocated to the combination ketamine and CBT/MM arm of this study will have the greatest reduction in pain intensity and pain interference.

#### Blinding Mechanism

The intervention delivery providers (anesthesiologists and psychologists) and other members of the research team will be aware of each participant’s assigned intervention. Within the research team, blinded members will be responsible for inputting all of the data into the REDCap (Research Electronic Data Capture; Vanderbilt University) database, with the exception of any ketamine-specific data, that is, semistructured interviews after the ketamine intervention and any adverse events. Following data collection, we will request blinded staff to record their “best guess” of each patient’s intervention to assess blinding efficacy. The research team currently has 1 blinded member who will serve as the outcome assessor and be responsible for data input.

#### Emergency Unblinding

To optimize study quality, emergency unblinding will only occur when knowledge of the intervention is essential for participant care, as determined by the principal investigator. In the case of emergency unblinding, the timing, reason for doing so, and personnel involved will be recorded in the case report form, while blinding is maintained by as many other study personnel as possible.

### Outcome Measures

#### Primary Outcome

The primary outcome of this pilot trial is to determine the feasibility of a large-scale multicenter trial. Feasibility outcomes will include the following variables, with all thresholds listed in parentheses:

1. Recruitment success (with the specific aim of recruiting all necessary patients within the first year of the trial period).
2. Adequate consent rate (>30% of eligible patients consent to participate in the study).
3. Percentage of patients who complete all medication and psychotherapy visits (ie, intervention adherence; >90%).
4. Percentage of patients who withdraw from the study (eg, due to side effects or other reasons; <20%).
5. Percentage of patients with incomplete pain intensity and pain interference data at 1 month and stated or ascribed reason (eg, withdrawal due to side effects, loss of follow-up, or missing data; <10%).
6. Rate of adverse outcomes (ie, 0 serious adverse events).

Based on the result for each primary feasibility outcome, we will determine whether the definitive study is feasible as proposed (ie, all feasibility targets are met), feasible with protocol modifications (modifications specified), or not feasible because feasibility targets could not be met even with protocol modifications. These cutoffs, identified in the parentheses above, will help determine whether protocol changes are required before proceeding with a definitive trial.

#### Secondary Outcome

The secondary outcomes chosen for the definitive trial follow consensus recommendations of core domains that should be addressed in studies examining chronic pain. Information will be collected in these domains as part of the pilot trial using survey-based outcome assessments, either in person during study visits or over the telephone by trained research personnel. The following pain score questionnaires will be used:

1. Pain intensity on an 11-point (0-10) Numeric Rating Scale.
2. Pain interference on the Patient-Reported Outcomes Measurement Information System (PROMIS-PI) scale.
3. Catastrophic thinking about pain, as measured by the Pain Catastrophizing Scale (PCS).
4. Features of depression, as measured by the Patient Health Questionnaire-9 (PHQ-9).
5. Features of anxiety, as measured by the Generalized Anxiety Disorder-7 (GAD-7).
6. Patient Global Impression of Change Scale (PGIC).

**Figure 1:**
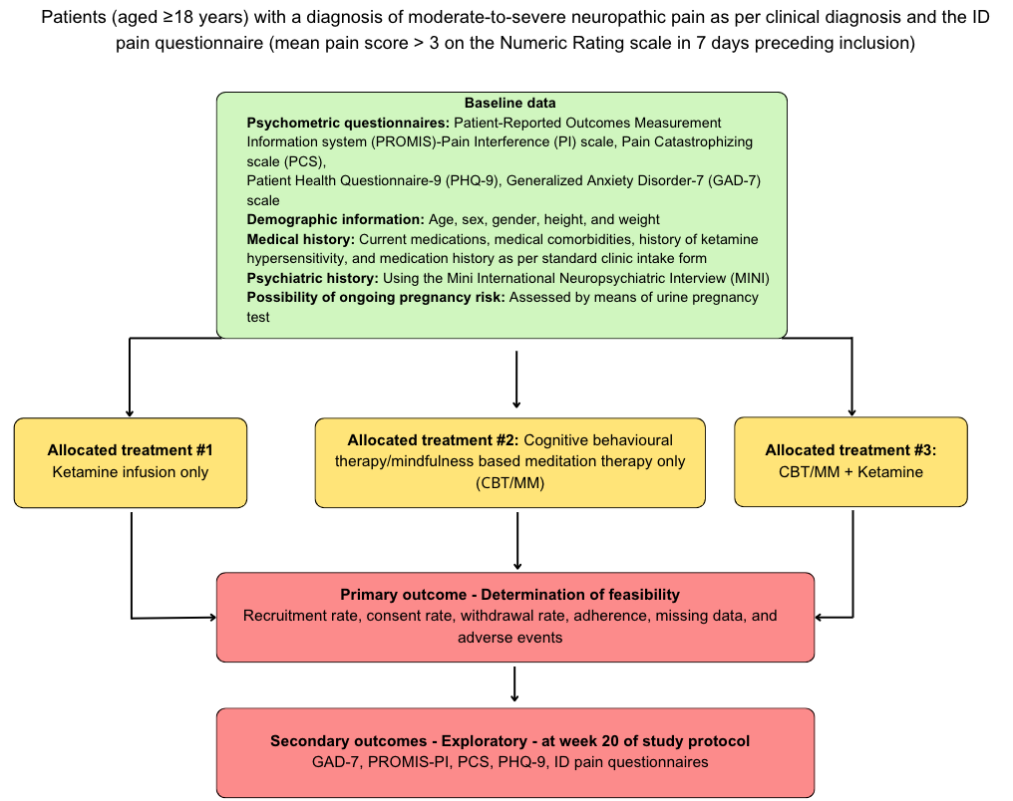
Overview of study design with primary and secondary outcomes. CBT/MM: cognitive behavior therapy/mindfulness and meditation.

Secondary clinical outcomes will include changes relative to baseline assessment in pain intensity and pain interference at week 20 (4 weeks post-completion of treatment; Figure 1). Scores for all outcomes at weeks 2, 7, 12, 16, and 20 will be compared to baseline scores to assess any progressive additive effects between the treatments. For any patients who withdraw before week 20, their last documented responses will be used as a comparison against baseline data. Patients will be allowed to adjust the dosages of other analgesic medications in conjunction with their primary physician. Analgesic medication use and doses will be tracked at the above time points to assess changes over time. Semistructured interviews will be conducted at 2 points within the study period: at weeks 1 and 20 and after the ketamine infusions (Table 1). The interviews will be audio-recorded to ensure the responses are accurately captured. In-person interviews will be recorded with an encrypted device, and phone interviews will be recorded through the Unity Health Zoom (Zoom Video Communications Inc) recording option. The audio recording files will be saved on St. Michael’s Hospital secure and restricted servers, and the files will be labeled by the study number. The recordings will be transcribed by the research team, and after verifying that the transcriptions are accurate, the audio recordings will be destroyed. Questionnaires will be delivered to patients at prespecified time points, as outlined in the study timeline below.

**Table 1:** Study timeline from weeks 1-20.

Time point		Screening	Week 1	Week 2	Week 7	Week 12	Week 16	Week 20
		Visit 1	Visit 2	Visit 3	Visit 4	Visit 5	Visit 6	Visit 7
**Enrollment**								
	Eligibility screen	✓						
	Informed consent	✓						
	MINI^a^	✓						
	Randomization		✓					
**Interventions**								
	Ketamine-hydrochloride infusion (arm 1 and arm 3)			✓	✓	✓		
	Remote CBT/MM^b^ (arm 2 and arm 3)		✓	✓	✓	✓	✓	
	In-person CBT/MM (arm 2 and arm 3)			✓	✓	✓		
**Assessments**								
	Baseline data		✓					
	Semistructured interviews		✓^c,d^	✓^d^	✓^d^	✓^d^		✓^c,d^
	Pregnancy test (if applicable)^e^			✓	✓	✓		
	Questionnaires: PROMIS-PI^f^, PGIC^g^, GAD-7^h^, PHQ-9^i^, and PCS^j^		✓	✓	✓	✓	✓	✓
	Adverse events			✓	✓	✓	✓	✓

^a^MINI: Mini International Neuropsychiatric Interview.

^b^CBT/MM: cognitive behavior therapy/mindfulness and meditation.

^c^Participants randomized to receive CBT/MM alone (arm 2), will receive a semistructured interview on weeks 1 and 20.

^d^Participants randomized to receive ketamine-hydrochloride infusion + CBT/MM (arm 3) will receive a semistructured interview on weeks 1 and 20 as well as a semistructured interview during the ketamine infusion on weeks 2, 7, and 12.

^e^Only female participants randomized to receive ketamine-hydrochloride alone (arm 1) or the ketamine-hydrochloride infusion + CBT/MM (arm 3) will require a pregnancy test on the day of infusion.

^f^PROMIS-PI: Patient-Reported Outcomes Measurement Information System.

^g^PGIC: Patient Global Impression of Change Scale.

^h^GAD-7: Generalized Anxiety Disorder-7.

^i^PHQ-9: Patient Health Questionnaire-9.

^j^PCS: Pain Catastrophizing Scale.

### Ethical Considerations

This study will be conducted in accordance with the ethical principles laid down in the Declaration of Helsinki, the protocol, Good Clinical Practice guidelines, and applicable regulatory requirements. This study received a “No Objection Letter” from Health Canada on October 13, 2022, indicating the study protocol was deemed acceptable.

Full written informed consent will be obtained from participants before conducting any study activities. The study was reviewed and approved by the Research Ethics Board at St. Michael’s Hospital on May 5, 2023 (22-217). This trial was designed and will be reported according to the CONSORT (Consolidated Standards of Reporting Trials) clinical trials statement checklist and the CONSORT-Outcomes 2022 extension [16].

## Results

### Prestudy Screening

Eligible participants will be screened at the Chronic Pain Clinic at St. Michael’s Hospital. Participants diagnosed with chronic neuropathic pain that meet the criteria from the ID pain questionnaire will be approached and asked to consider study participation.

After participants have provided informed consent, they will be asked about their medical comorbidities, psychiatric history as based on the Mini International Neuropsychiatric Interview, history of ketamine hypersensitivity, and concomitant medications to confirm eligibility. If a participant is eligible to participate in the study, the participant will be enrolled in the study, and the study coordinator will book an appointment for the participant’s study intervention.

At this current point, November 1, 2023, the study has not started enrolling and consenting patients.

### Baseline Data

After initial screening and informed consent are obtained, the following data will be collected at baseline (week 1):

1. Demographic information: age, sex, gender, height, and weight.
2. Detailed data regarding current medications and medical comorbidities, as well as medication history, is collected by our standard intake form.
3. Chronic neuropathic pain severity and other relevant covariates will be assessed at baseline using:
  - PROMIS-PI
  - PCS
  - PHQ-9
  - GAD-7
  - PGIC

### Follow-Up Data

In all arms, at each follow-up visit, participants will complete the PROMIS-PI scale, PCS, PHQ-9, GAD-7, and the PGIC to assess outcome measurements. Follow-ups will be conducted every 4 weeks over the phone to maintain the blindness of the research team.

For females, a urine pregnancy test will be required for those randomized to the ketamine-hydrochloride infusion alone or the ketamine-hydrochloride infusion+CBT/MM arms in order to confirm that the patient is not pregnant before each ketamine infusion treatment.

### Outcome Analysis

Based on the primary end points, we will determine whether or not this pilot study is as follows:

1. Feasible: all feasibility outcomes are met; no protocol modifications are needed,
2. Feasible with modification: all feasibility outcomes are met or can be met with protocol modifications, and
3. Not feasible: Even with protocol modifications, some feasibility outcomes cannot be met.

In terms of statistical analyses for this feasibility study, preliminary analyses will consist of univariate tests to compare clinical and demographic variables of interest. Linear regression analysis will be used to quantify the strength and magnitude of the relationship between the intervention and the coprimary outcomes. Secondary outcomes will be analyzed as continuous measures using generalized linear regression models.

For any missing data where patients do not attend the allocated sessions or do not complete all components of the relevant questionnaires, this will be noted and documented to assess the overall feasibility of this study. Data will not be extrapolated or interpreted for these missing data sets.

### Study Status As of Date

As of the current date, November 1, 2023, the study has not started to recruit patients.

## Discussion

It is hypothesized that concurrent pharmacotherapy and psychotherapy have combined effects on the analgesic response. In addition to ketamine’s anesthetic-analgesic properties, it also uniquely alters the individual’s level of consciousness at variable doses. Some researchers posit that the therapeutic experience of psychotherapy may be augmented by ketamine’s alteration of conscious awareness [10]. As demonstrated by this systematic review, few studies have examined ketamine-assisted psychotherapy (KAP) for chronic neuropathic pain. RCTs have not been published on this topic. In addition, there appears to be wide variability in the indication, dosage, and patient treatment setting, such that the current literature is inconclusive.

The 3-arm design allows us to compare the feasibility of KAP with ketamine alone and CBT/MM. We will track feasibility outcomes, including recruitment rate and adverse event rate, to estimate the acceptability of this intervention compared to controls. There are also limitations to this study. Due to the participatory nature of CBT/MM, we are unable to blind patients and the research team to patients’ assigned interventions. To mitigate this, the outcome assessors will be blinded to minimize bias, and we will request and report their “best guess” estimate of each patient’s assigned intervention at the end of the study. We also recognize that there may be different patient outcomes based on the mode of CBT/MM delivery. Patients in arm 1 receiving ketamine alone will have no CBT/MM as part of the trial design. Patients assigned to CBT/MM alone (Arm 2) will be receiving their intervention sessions remotely rather than in-person for 16 weeks. Patients in Arm 3 will be receiving ketamine on weeks 2, 7, and 12 and in-person CBT/MM at this time. They will also be involved with remote CBT/MM sessions from weeks 1 to 16 (except for weeks 2,7, and 12). This hybrid intervention will allow us to collect data on the feasibility of in-person versus remote psychotherapy for this patient population.

This study will inform the development of a larger-scale multicenter RCT. Our results will help inform the sample size calculation to ensure an adequately powered, definitive RCT. We anticipate that the psychotherapy and ketamine protocols developed in this study will be easily replicable and scalable should they show clinical and statistical benefits in the chronic neuropathic pain population. Furthermore, future studies developed by our research team will aim to study longer-term outcomes associated with KAP and the need for ongoing therapy in this patient subset.

## Appendix

Multimedia Appendix 1 Peer review report from the Canadian Pain Society in partnership with the Pfizer Early Career Investigator Award.

## Abbreviations

CBT: cognitive behavioral therapy
CONSORT: Consolidated Standards of Reporting Trials
GAD-7: Generalized Anxiety Disorder-7
KAP: ketamine-assisted psychotherapy
MM: mindfulness and meditation
PCS: Pain Catastrophizing Scale
PGIC: Patient Global Impression of Change Scale
PHQ-9: Patient Health Questionnaire-9
PROMIS-PI: Patient-Reported Outcomes Measurement Information System
RCT: randomized controlled trial
REDCap: Research Electronic Data Capture
