# Supplementary material for: Psychotherapy for Ketamine’s Enhanced Durability in Chronic Neuropathic Pain: Protocol for a Pilot Randomized Controlled Trial
Source: JMIR Res Protoc. 2024 Apr 17;13:e54406. doi: 10.2196/54406 (PMC11063874; doi:10.2196/54406)
Supplement: Multimedia Appendix 1 [file resprot_v13i1e54406_app1.pdf]

the CANADIAN PAIN SOCIETY
la SOCIÉTÉ CANADIENNE de la DOULEUR

Pfizer

20 Crown Steel Dr, Markham, Ontario L3R9X9 • cps@secretariatcentral.com • www.canadianpainsociety.ca • +1-365-873-2320

April 14, 2022

CPS/Pfizer EARLY CAREER INVESTIGATOR PAIN RESEARCH GRANT PROGRAM 2022

Dear Dr. Goel,

Congratulations! On behalf of the Canadian Pain Society and Pfizer Canada, I am pleased to inform you that your application entitled “Psychotherapy for Ketamine’s Enhanced Durability in Chronic Neuropathic Pain - PSYKED-NP” has been approved for funding. The award start date is June 1, 2022.

The competition was extremely competitive this year and you should take great pride in this achievement. Appended to this letter you will find detailed reviewer comments that we hope will helpyou as you conduct your research.

As a recipient of this grant, you will receive $50,000 CAD to support operating costs associated with pilot or preliminary research that can be used as the basis for a tri-council grant submission. The CPS office will arrange for payment of your award, so please provide contact information for the person responsible for grant and finance management at your institution.

The duration of this grant is for one year - however if, for any reason, you require an extension an additional year can be provided upon request. Should any funds remain unspent at the end of that period, they will need to be returned to the Canadian Pain Society.

Awardees will be expected to acknowledge CPS/Pfizer funding in all publications and presentations resulting from the funded research, submit a written report to the awards committee at the end ofthe grant and submit your research for presentation at the CPS Scientific meeting within 2 years ofcompletion.

Contact information for a finance person and extension requests (if necessary) should be sent to the awards committee chair at cps@secretariatcentral.com.

Finally, if you no longer meet the eligibility criteria (e.g. have received a major operating grant as PI or Co-PI that provides yearly support equal to or greater than the CPS/Pfizer Early Career Research Grant since initial submission, you must notify us immediately.

Congratulations on your success in this competition! We look forward to seeing you in the future!

Sincerely,

Lynn Gauthier

Lynn Gauthier, PhD
CPS Awards Committee Chair
Assistant Professor
Fonds de Recherche du Québec - Santé Research Scholar Junior 1-In partnership with the Québec Breast Cancer Foundation
Department of Family and Emergency Medicine
Chair, Palliative Care Research
Faculty of Medicine, Université Laval
Scientist, L’Équipe de Recherche Michel-Sarrazin en Oncologie psychosociale et Soins palliatifs
CR-CHU de Québec, oncology division & Cancer Research Centre, Université Laval

20 Crown Steel Dr, Markham, Ontario L3R9X9 • cps@secretariatcentral.com • www.canadianpainsociety.ca • +1-365-873-2320

Reviewer comments

Strengths of the application

Reviewer1

- Addressing clear gap in existing research with obvious immediate clinical relevance.
- Strong use of early career grant for pilot feasibility RCT to establish case for definitive trial with
- larger funding applications.
- Applicant has strong publication record (>25 peer-reviewed articles), research success, and
- several leadership awards.
- Strong collaborative and mentorship team, and access to established research group
- Supportive reference letters ("an exciting academic visionary with the ability to collaborate
- across specialties and arenas").

Reviewer 2

- This study is the first step in an extremely important line of research that will have very important clinical application. The PI has an established interest in the field (published systematic review). The investigators are already implementing the components of the intervention clinically and now propose to combine them in a clinical trial to test the synergy between the psychological (CBT) intervention and ketamine to prolong the treatment effects of ketamine.

Reviewer 3

- Applicant has training in epidemiology and health care research and has published several impactful papers.
- Proposal
- Interesting research question to determine whether a ketamine-induced neuroplastic state can make CBT more effective and potentially will provide more sustained ketamine-induced pain relief. Although this is a pilot RCT, it could eventually answer an important research question that could improve mental health and quality of life in these refractory pain patients
- The PI has performed a systematic review of previous research in area of proposed study.
- Justified rationale
- Strong multidisciplinary research team with fantastic track record in publications and trials. Patient representative included
- Objective outcome measurements (Fitbit)
- Realistic budget

Weaknesses of the application

Reviewer1

- Project proposal would be strengthened by using pilot feasibility RCT headings for methods.
- Some relevant information missing including rationale for sample size of n=10/group (total
- n=30) for pilot RCT goals and for identified benchmarks for determining feasibility domains,
- and methods for randomization.
- Minimal detail on healthcare provider participants in post-trial qualitative interviews. Unclear
- who will be interviewed, how this will inform future definitive trial and/or be combined with
- quantitative findings in pilot RCT.
- A primary rationale for the larger definitive trial is to examine longer term effects of combined

20 Crown Steel Dr, Markham, Ontario L3R9X9 • cps@secretariatcentral.com • www.canadianpainsociety.ca • +1-365-873-2320

- ketamine and psychological interventions (i.e., >12 months); however, the pilot RCT is only 16 weeks duration. It is unclear how lessons learned from this shorter feasibility study will inform
- successful design of a definitive trial of a longer duration (e.g., potential participant attrition, timing of ketamine infusions).
- Inclusion of a patient partner / person with lived experience; yet, unclear how they will
- meaningfully inform trial methods and outcomes since those are already outlined. Problematic that no compensation is listed in budget for the patient partner.
- Are benefits included in the budget for the clinical research coordinator?

Reviewer 2

I have a few suggestions for the investigators to consider.

- In any grant proposing a clinical trial, it is helpful to include a study schema to help orient the reader; I strongly suggest it for your future applications.
- The way I read your proposal is the goal of the study is to test whether adding CBT to ketamine will increase the duration of effect. If this is the case (and you aren't going to include a placebo group), why not just use an add-on design "(i.e., ketamine alone vs. ketamine +CBT). This would decrease the complexity of the study and number of groups, all of which will make an eventual trial much easier to accomplish. If you also are interested in understanding how ketamine alone vs CBT alone works, etc. then it make sense to include the CBT alone, but then you should discuss why this is an important question as well in your background.
- I appreciate your rationale for not including a placebo only group or usual care, but that does not preclude you from including a placebo infusion in the CBT group and an attention control (e.g., health education) in the ketamine infusion only group. I would justify excluding these types of controls.
- It is not clear for which timepoint your feasibility analyses will be made. I assume 20 weeks, but this should be clarified.
- Your feasibility goals seem a bit idealistic to me. 10% withdrawal rate is quite low for a chronic pain RCT. Also, if you have 10% withdrawal rate, do you really expect to have only 5% missing data? This assumes that at least half of the people who withdraw will be willing to still participate in assessments (if your withdrawal rate is in fact 10%).
- The definitions of your feasibility data should be better defined. For example, when you say missing data, do you mean the primary clinical outcomes at the final timepoint or all outcomes at all timepoints? You need to better define what you mean by intervention adherence >90%. For example, does that mean that >90% of participants will complete EVERYTHING or that EVERYONE will complete >90% of CBT and infusions?
- 30 patients at one site in a year for a fairly long RCT is a bit ambitious, but since that is one of the outcomes, I think this is ok.

Reviewer 3

- Pilot RCT, no effectiveness/efficacy trial
- Study population: ID questionnaire is used to diagnose neuropathic pain but has lower sensitivity and specificity
- Study population: Although planned as feasibility study, you are measuring clinical outcomes. Therefore, there are some demographics that might be worth tracking due to their potential impact on clinical outcome: duration of pain, etiology of neuropathic pain (post-herpetic, post-traumatic)
- Contra-indications: Improvement of mood/mental health is an off-label indication for ketamine. Hence, Health Canada approval will likely be required to start this study. This will have its impact on the study time line, broader exclusion criteria (hypertension, hyperthyroidism, impaired liver/kidney

20 Crown Steel Dr, Markham, Ontario L3R9X9 • cps@secretariatcentral.com • www.canadianpainsociety.ca • +1-365-873-2320

function, ..) and recruitment.

- Intervention: Please consider dosing of ketamine according to weight (eg 0.5 mg/kg/h) instead of a fixed amount (80 mg) to measure an unbiased treatment effect/ incidence of adverse outcomes.
    - The analgesic medication can be adjusted by the primary physician who will be blinded to treatment allocation. If the patient is not blinded to allocation, how can the PI ensure the family physician will be?
    - Not placebo-controlled: Has the PI considered a fourth arm with saline infusions + CBT ? This could be added and sedating medication (midazolam) can be provided to both infusion groups to enhance blinding.
- Patients are not blinded to treatment allocation. Hawthorne effect
